# Variation in the use of percutaneous coronary interventions among older patients with acute coronary syndromes: a multilevel study in Fukuoka, Japan

**DOI:** 10.1186/s12939-021-01415-4

**Published:** 2021-03-16

**Authors:** Yunfei Li, Akira Babazono, Aziz Jamal, Takako Fujita, Shinichiro Yoshida, Sung-A Kim

**Affiliations:** 1 Department of Health Care Administration & Management, Graduate School of Medical Sciences, Kyushu University, Fukuoka, Japan; 2 Health Administration Program, Faculty of Business & Management, Universiti Teknologi MARA, Selangor, Malaysia; 3 Department of Health Sciences, Faculty of Medical Sciences, Kyushu University, Fukuoka, Japan

**Keywords:** Percutaneous coronary intervention, Multilevel analysis, Small area variation, Hospital variation, Japan

## Abstract

**Background:**

Variation in health care delivery among regions and hospitals has been observed worldwide and reported to have resulted in health inequalities. Regional variation of percutaneous coronary intervention (PCI) was previously reported in Japan. This study aimed to assess the small-area and hospital-level variations and to examine the influence of patient and hospital characteristics on the use of PCI.

**Methods:**

Data provided by the Fukuoka Prefecture Latter-stage Elderly Insurance Association was used. There were 11,821 patients aged ≥65 years with acute coronary syndromes who were identified from 2015 to 2017. Three-level multilevel logistic regression analyses were performed to quantify the small-area and hospital variations, as well as, to identify the determinants of PCI use.

**Results:**

The results showed significant variation (δ^2^ = 0.744) and increased PCI use (MOR = 2.425) at the hospital level. After controlling patient- and hospital-level characteristics, a large proportional change in cluster variance was found at the hospital level (PCV 14.7%). Fixed-effect estimation results showed that females, patients aged ≥80 years old, hypertension and dyslipidemia had significant association with the use of PCI. Hospitals with high physician density had a significantly positive relationship with PCI use.

**Conclusions:**

Patients receiving care in hospitals located in small areas have equitable access to PCI. Hospital-level variation might be originated from the oversupply of physicians. A balanced number of physicians and beds should be taken into consideration during healthcare allocation. A treatment process guideline on PCI targeting older patients is also needed to ensure a more equitable access for healthcare resources.

## Introduction

Variation in healthcare delivery among regions and hospitals has been observed worldwide. It has been posited that these variations in healthcare delivery can be explained by patient demographics and by the influence of healthcare supply that is characterized at the regional and hospital levels. In the early 1960s, a positive association between the supply of hospital beds and hospitalization rates was demonstrated [1]. In 1973, the first study on small-area variations in health care delivery reported significant small-area variations in many aspects of health care, including the relationship between physician distribution and a range of service provisions [2]. The authors noted that areas with more surgeons would provide more surgeries. Since then, studies have consistently suggested that there is variation in, and that geographical characteristics contribute to, health-care delivery and physician preference in clinical decision-making.

Percutaneous coronary intervention (PCI) is one of the procedures that strongly depends on physicians’ preferences and recommendations, and its performance is commonly used to address acute coronary syndrome [3, 4]. However, its efficacy has been somewhat debated in the past decades. Some clinical trials conducted from Japan showed conflicting founding. Although improved long-term efficacy was demonstrated in one study [5], another study found no significant difference in the risk of any major cardiovascular events between PCI and medical therapy [6]. Further research did not find its cost-effective advantages for the addition of PCI [7]. Despite of its unclear clinical outcomes and economic benefits, the number of PCI procedures has been increasing over the past decade. It is estimated that more than 200,000 PCI procedures are performed annually in Japan, and older adults accounted for a large proportion [8]. To meet the growing demand for PCI, the Japanese Circulation Society has issued several PCI guidelines to provide useful information for institutions and physicians during treatment process [9, 10]. However, as a special group, the decision to use PCI for older adults is more complicated than for younger people [11, 12].

Patient’s decision to undergo PCI is mainly influenced by two factors: 1) the patient’s ability to pay for PCI; and 2) patient’s expectation which is led by physicians’ preferences and recommendations [13]. Patients would expect the best health outcome at a minimum cost. Under universal health care scheme, older patients only need to pay a small fraction of the high medical expenses in Japan [14], the equitable access to PCI can be ensured. To the hospitals, PCI procedure is relatively profitable as the total cost for PCI per person could be as high as US$10,000 [15], and hospitals could receive an additional incentive of approximately US$3500 for an urgent PCI from the government [16]. Consequently, such a procedure provides an economic incentive for hospitals and physicians to recommend patients to undergo PCI. Therefore, both patients and hospitals have incentives to opt for PCI when addressing acute coronary syndromes. These incentives will introduce unwarranted variations for the provision of health care delivery in the long run.

Some studies have investigated the understanding of regional and hospital-level variations in the use of PCI worldwide [17–20]. It is reported that regional variation in PCI has been observed in England. This variation was unexplained by procedure volume or deprivation. Instead, practitioner preference was considered as the contribution of unwarranted influences [18]. Regional variation in PCI use was also reported in hospitals across China. The variation linked to both geographic locations and hospital characteristics. The improvement for the quality of PCI in those regions, such as the management for high-risk patients, physicians’ qualification and skills, and knowledge sharing on PCI technology was needed [19]. A recent study conducted in the US demonstrated the presence of disparity in PCI use in high-risk population across all geographic regions. This regional variation could be explained by both patient and hospital characteristics. Targeted improvement in the application of advanced evidence-based therapies among high-risk population are recommended [20]. In Japan, regional variation in the use of PCI had been reported nationwide [17]. The study reported that PCI performed more than was needed in regions with higher PCI rates from the hospital perspective. However, the reason of this variation has not been thoroughly investigated due to the lack of information regarding patient characteristics. Besides, the variation in PCI use between and among hospitals were not examined among small areas.

Based on this fact, we are motivated to conduct this study to examine the variations on PCI use within small area and among hospitals, and to investigate the determinants of PCI use at both patient and hospital levels in Japan. In this study, we extracted data from a claims database, which includes the information of patient and hospital characteristics, and conducted three-level multilevel analyses on PCI use among older patients with acute coronary syndromes to examine the possible variation across small areas and hospitals. We further examined the influence of patient and hospital characteristics on PCI use to identify the factors that would potentially play significant roles in explaining variations in this widely performed medical practice. The findings of this study might provide useful information for policymakers, local government agencies, and hospital administrators in designing policies for better allocation of acute healthcare resources, and to develop health management strategies for older people.

## Methods

### Study design and data source

This study was designed as an observational study. We obtained data from the Latter-stage Elderly Healthcare Insurance Association of Fukuoka Prefecture, Japan. This public insurance association provides policy to residents aged ≥75 years and residents aged between 65 and 74 years with a specified disability. Information about sex, birth date, residence region, economic status, and diagnostic and treatment procedures are available in the database. Data were extracted for 2 fiscal years (i.e., 1 April 2015 to 31 March 2017).

In this study, we defined acute angina and acute myocardial infarction (AMI) as acute coronary syndromes because these conditions were considered to be clinical indications for PCI. *International Classification of Diseases 10th Revision* (ICD-10) codes were used to determine MI and angina diagnoses. ST-segment elevation myocardial infarction (STEMI) was defined by ICD-10 codes I21.0—I21.3, non-ST-segment elevation myocardial infarction (NSTEMI) by I21.4, and unspecified AMI by I21.9. Unstable angina was defined by ICD-10 codes I20.0, stable angina by I20.1 and I20.8, and unspecified angina by I20.9. The type of diagnosis procedure combination (DPC) in the database (whether DPC or not) was used to distinguish patients’ acute conditions. DPC was introduced in 2002 to contain health expenditures and to improve the quality of care in Japanese care facilities to cover most acute in-patient care [21]. DPC data include anonymous charge data, clinical data, and care-process data. We identified participants with a diagnosis of acute coronary syndromes. Subsequently, we identified patients who underwent PCI and excluded those with the records of coronary artery bypass grafting (CABG). In Japan, CABG is not regarded as the first choice by the physicians to address the coronary artery disease and the number of CABG procedures performed is quite small.

Economic status in the insurance database is categorized into low, middle, and high. These categories are applied by insurance associations operated in all prefectures in Japan. Such categories also correspond to the threshold set by the Japanese Ministry of Health, Labour and Welfare based on the annual income: low-level, less than 1,550,000 yen ($13,868.5); middle-level, 1,550,000—3,830,000 yen (($13,868.5—34,268.7), high-level, more than 3,830,000 yen ($34,268.7). In Fukuoka Prefecture, statistics collected in 2017 indicate 48.6% of population over 75 years old belongs to middle-income category, whereas the 45.7% of the population categorized in middle-income group. The remaining 5.7% belongs to the high-income category. The category of low income is further extended into two groups namely low and very low. The annual income less than 800,000 yen ($7157.0) is considered very low income.

Charlson Comorbidity Index (CCI) scores were used to assess the severity of comorbidity status. Previous studies showed that the CCI has acceptable reliability, and such an index could be applied to studies using the Japanese insurance claims database [22, 23]. We determined the CCI using 17 reported comorbidities (AMI, congestive heart failure, peripheral vascular disease, cerebral vascular disease, dementia, chronic pulmonary disease, connective tissue disorders, peptic ulcer, liver disease, diabetes, diabetes complications, paraplegia, renal disease, cancer, metastatic cancer, severe liver disease, and HIV). ICD-10 coded data were used to capture these morbidities. These comorbidities were assigned weights ranging from one to six to assess their potential impact on treatment prognosis and mortality [24]. In this study, we modified the CCI scores by excluding the AMI diagnosis. The modified CCI scores for each patient were calculated by adding the weights of these comorbidities. Because several clinically important diagnoses are directly assessed by the CCI, we extracted hypertension (ICD-10 code: I10-I15) and dyslipidemia (ICD-10 code: E781-E785) separately.

**Fig. 1:**
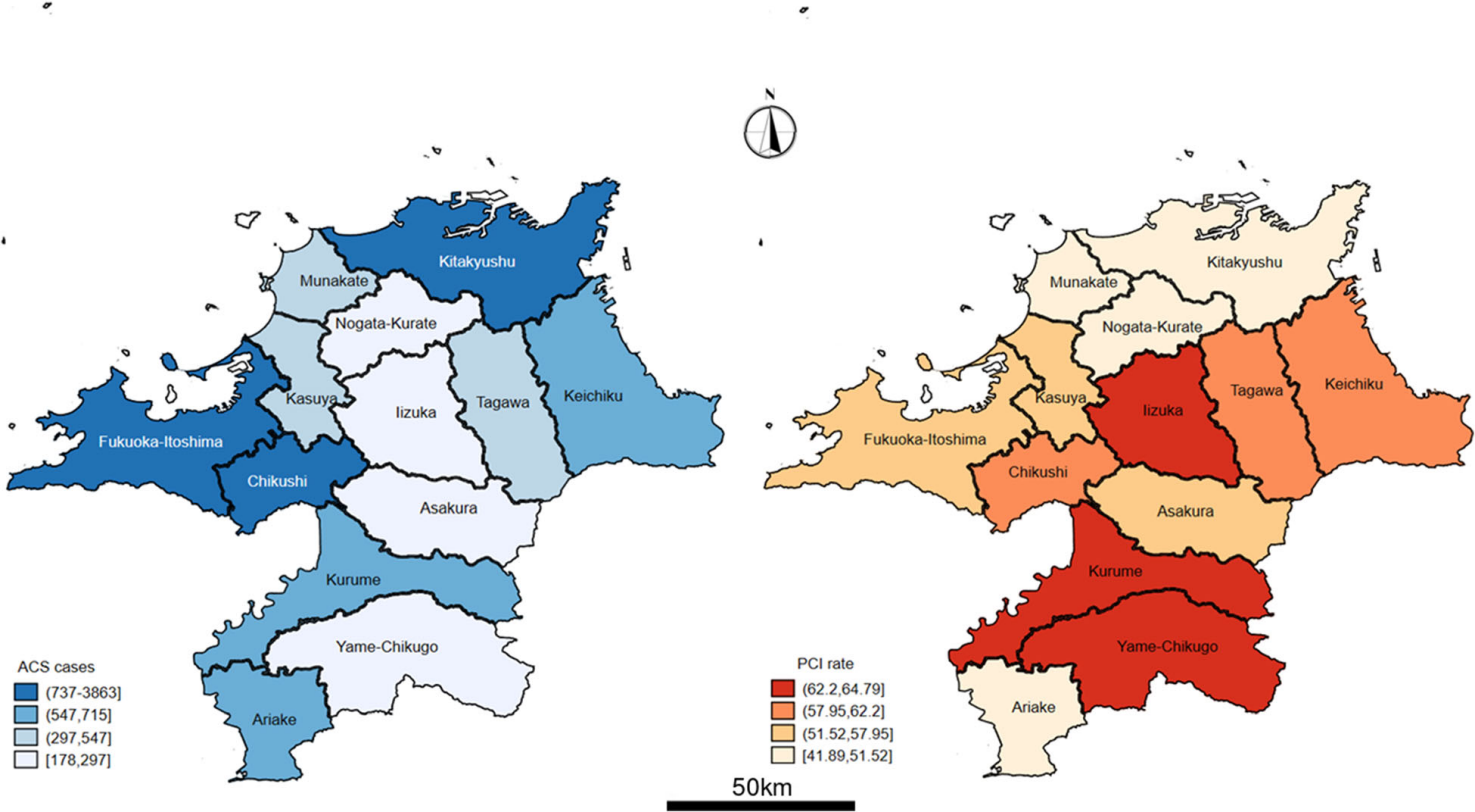
Patients with ASC by SMA in Fukuoka Prefecture, Japan (left column) and PCI rates among SMA in Fukuoka Prefecture, Japan (right column). ASC, acute coronary syndrome; PCI, percutaneous coronary intervention; SMA, secondary medical area

Information about the number of beds, hospital ownership, and number of physicians at the hospitals was extracted from the database. Information from 84 hospitals was extracted from the database. Hospitals providing PCI were categorized by “secondary medical area” (SMA), which represents the unit care service provision that is governed by each prefecture in accordance with Japan’s Medical Service Law. Thirteen SMAs were defined in Fukuoka Prefecture [25]. Figure 1 (left column) depicts the number of patients with acute coronary syndrome by SMA in Fukuoka Prefecture. Data extraction was performed using SQL Server 2014 programming language (Microsoft Corporation, Redmond, WA, USA).

On the basis of the Japanese government report, the insurance databases had a high penetration rate—approximately up to 98.6% until April 2015 [26]. Japanese Health Insurance Claims Review & Reimbursement services are responsible for the quality control of computer-administered claims databases. We used anonymized claims insurance data, and therefore, informed consent was not required. The study was approved by the Institutional Review Board at Kyushu University (Clinical Bioethics Committee of the Graduate School of Medical Sciences, Kyushu University).

### Variables

The outcome variable is the PCI status—whether patients had PCI or not. Patients with acute coronary syndromes might receive PCI in combination with medication therapy or only medication therapy. Therefore, in this study, we defined patients who received PCI in combination with medication therapy as PCI cases, and patients who only received medication therapy as no PCI cases.

The explanatory variables were categorized into the following two groups: patient-level characteristics and hospital-level characteristics.

We identified information on sex, age, economic status, comorbidities, and CCI scores as patient-level characteristics. We categorized age into the following four groups: 65–74 years, 75–79 years, 80–85 years, and ≥ 85 years. The acute coronary syndrome was grouped into the following categories: 1) STEMI/unstable angina; and 2) other MI/angina conditions. STEMI/unstable angina are considered to be more severe conditions among patients with acute coronary syndrome. Economic status was classified into the following three groups: low-, middle-, and high-level. CCI scores were applied to define the severity of comorbidity conditions as follows: none (CCI = 0); mild (CCI = 1–2); moderate (CCI = 3–4); and severe (CCI = 5–higher) [27]. Other clinically important comorbidity status was also included as follows: hypertension and dyslipidemia.

We used the number of beds, ownership, and the physician density at hospitals as the hospital-level characteristics. Ownership was categorized as either public or private. The number of beds was categorized into the following three groups: ≤199, 200–399, and ≥ 400. According to the Japan’s Medical Service Law, a regional medical care support hospital needs to have at least 200 beds, and an advanced treatment hospital needs to have at least 400 beds. Physician density was defined as the number of full-time physicians per bed in each hospital. Because optimal physician density was not known, we classified physician density into 3 tertiles: the first, the second, and the third tertiles. The first tertile presents hospitals with comparatively small number of physicians and excessively high number of hospital beds. The third tertile represents hospital with excessively high number of physicians and comparatively small number of hospital beds. The second tertile consists of hospitals with a balanced ratio of physicians and beds.

### Statistical analysis

We calculated the number of patients with acute coronary syndrome, the number and percentage of PCI, and hospital physician density by SMA. We then calculated the number of PCI on the basis of patient and hospital-level characteristics. The relationship between PCI and these characteristics was quantified using the univariate odds ratio (OR) with a 95% confidence interval (CI).

To quantify the magnitude of regional and hospital variations, as well as to identify determinants on the use of PCI, we fitted three-level multilevel logistic models with random intercept, setting patients as level 1, hospitals as level 2, and SMAs as level 3. In this study, we specified the models for PCI use with patients *i* nested in hospitals *j* that are nested in SMA *k*. This multilevel model can be expressed using the following equation [28–30]:

1 $$ logit\ \left\{\Pr \left({y}_{ijk}=1|{x}_{ijk},{\tau}_{jk}^{(2)},{\tau}_k^{(3)}\right)\right\}={\beta}_{000}+{\sum}_{i=1}\left({\beta}_{ijk}\ast {x}_{ijk}\right)+{\sum}_{j=1}\left({\beta}_{0 jk}\ast {x}_{jk}\right)+{\sum}_{k=1}\left({\beta}_{00k}\ast {x}_k\right)+{\tau}_{jk}^{(2)}+{\tau}_k^{(3)}. $$

In Eq. (1), *x*_*ijk*_ is a vector containing all covariates. The notation ${\tau}_{jk}^{(2)}$ represents a random intercept varying over hospitals (level 2), and ${\tau}_k^{(3)}$ is a random intercept varying over SMAs (level 3). The random intercept ${\tau}_{jk}^{(2)}$ and ${\tau}_k^{(3)}$ are assumed to be independent of each other and independent across SMAs, and ${\tau}_{jk}^{(2)}$ is assumed to be independent across hospitals. Both random intercepts are assumed to be independent of the covariates *x*_*ijk*_. Additionally, *β*_000_ + ∑_*i* = 1_(*β*_*ijk*_ ∗ *x*_*ijk*_) + ∑_*j* = 1_(*β*_0*jk*_ ∗ *x*_*jk*_) + ∑_*k* = 1_(*β*_00*k*_ ∗ *x*_*k*_) refers to the fixed part of the model and does not include random effects. *β*_000_ is the average intercept, *β*_*ijk*_ is the regression coefficient of the patient-level characteristics, *β*_0*jk*_ is the regression coefficient of the hospital-level characteristics, and *β*_00*k*_ is the regression coefficient of SMA-level characteristics, while *x*_*ijk*_ is the variable for patient-level characteristics, *x*_*jk*_ is the variable for hospital-level characteristics, and *x*_*k*_ is the variable for SMA-level characteristics.

We first fitted a null model only with random effect to estimate variations in PCI between hospitals and between the SMA level. We then developed model 1 by including patient-level characteristics into the null model. We subsequently developed model 2, inputting both patient and hospital-level characteristics into the null model to detect contextual effects for PCI use. In these models, we calculated area level variance (δ^2^), intraclass correlation coefficient (ICC) for similarity within groups, median odds ratio (MOR) for variance between groups, and proportional change in variance (PCV) for each level.

ICC can be interpreted as the variation between hospitals/SMAs, which can be considered to be an important measure of homogeneity of PCI use within hospitals/SMAs [31, 32]. The formula for calculating ICC was taken from a previous study [33], as follows:

2 $$ ICC=\frac{\delta^2}{\delta^2+{\pi}^2/3}. $$

MOR can be defined as the median odds ratio between the patients who are at a higher risk of the outcome and the patients at the lower risk of the outcome, assuming the same patient covariates from different hospitals/SMAs. The measure normally takes a value greater than 1. There would be no variation between clusters (hospitals/SMAs) if MOR equals 1. If there were considerable between-cluster variation, the MOR would be larger [34], which means that the variation in the hospital/SMA level largely exists. The equation for MOR is evaluated as follows [34]:

3 $$ MOR=\exp \left(\sqrt{2\times {\delta}^2}\times {\varPhi}^{-1}(0.75)\right), $$

where Φ^−1^(0.75) =0.6745 represents the 75th percentile of a standard normal distribution.

PCV estimates the proportional change in between-cluster (hospital/SMA) variance that is explained by introduction of additional patient and/or hospital characteristics [33, 35]. The equation for PCV is as follows [35]:

4 $$ PCV=\frac{\left({\delta}_i^2-{\delta}^2\right)}{\delta_i^2}, $$

where ${\delta}_i^2$ is the variance of the initial (null) model, and δ^2^ is the variance of the model with patients and/or hospital characteristics.

The Akaike’s information criterion (AIC) and log-likelihood were estimated to assess the models’ goodness-of-fit.

### Sensitivity analysis

We constructed several models to ensure the robustness of the analyses. Model 3 was constructed by replacing quartiles with continuous CCI scores. The age group in model 4 was replaced by continuous age. Model 5 was constructed by replacing the economic status in the model with quartiles (very low, low, middle, and high level).

All reported *P* values were two-tailed, and the level of significance was set at *P* < .05. All statistical analyses for this study were performed using Stata Statistical Software: Release 15 (Stata Corp LLP, College Station, TX, USA).

## Results

### Descriptive analysis

The descriptive statistics of patients with the acute coronary syndrome, patients who received PCI, and the average number of physicians at 84 hospitals in 13 SMAs are shown in Table 1. PCI rates that are represented by SMAs are shown in Fig. 1 (right column). Overall, among 11,821 patients with the acute coronary syndrome, 6420 (54.31%) patients had received PCI. Kitakyushu and Fukuoka-Itoshima had more patients who received PCI than other SMAs. The number of patients with PCI status in Kitakyushu and Fukuoka-Itoshima were 1602 and 1888, respectively. PCI rates were among the lowest in Ariake (41.89%) and Kitakyushu (48.87%), and the rates in Yame-Chikugo (64.79%) and Iizuka (64.79%) were among the highest. For the 84 hospitals that performed PCI, most of them were located in Fukuoka-Itoshima (*n* = 26) and Kitakyushu (*n* = 20). The highest average number of physicians was in Kurume (185.9), Iizuka (175.8), and Kitakyushu (138.4). The ratio of the highest to the lowest average number of physicians was approximately 11.6.

**Table 1:** Patient and hospital information on the basis of SMA

SMA	Total	PCI	No PCI	Number of hospitals	Average number of physicians
		N (%)	N (%)		
*Fukuoka-Itoshima*	2878	1602 (55.66)	1276 (44.34)	26	136.8
*Kasuya*	547	317 (57.95)	230 (42.05)	3	80.1
*Munakata*	474	240 (50.63)	234 (49.37)	2	51.5
*Chikushi*	737	449 (60.92)	288 (39.08)	3	133.0
*Asakura*	178	96 (53.93)	82 (46.07)	1	33.0
*Kurume*	715	457 (63.92)	258 (36.08)	11	185.9
*Yame-Chikugo*	213	138 (64.79)	75 (35.21)	4	55.9
*Ariake*	549	230 (41.89)	319 (58.11)	6	15.9
*Iizuka*	267	173 (64.79)	94 (35.21)	2	175.8
*Nogata-Kurate*	297	153 (51.52)	144 (48.48)	2	19.6
*Tagawa*	521	315 (60.46)	206 (39.54)	2	39.0
*Kitakyushu*	3863	1888 (48.87)	1975 (51.13)	20	138.4
*Keichiku*	582	362 (62.20)	220 (37.80)	2	40.4
Total	11,821	6420 (54.31)	5401 (45.69)	84	127.9

*PCI* percutaneous coronary intervention, *SMA* secondary medical area

### Univariate analyses

The results of patient and hospital-level characteristics and the OR for PCI are shown in Table 2. These results indicate that the use of PCI among women was significantly lower than that among men (OR 0.69, 95% CI 0.64–0.74, *P* < .001). Patients in the age group ≥85 years were less likely to undergo PCI than patients in other age groups (OR 0.69, 95% CI 0.60–0.79, *P* < .001). The higher the patients’ economic status, the higher the ORs that were observed. Patients with STEMI or unstable angina have higher possibility to receive PCI. Significant increases in PCI use were observed among patients with hypertension and dyslipidemia. PCI is significantly related to the CCI status severity. For hospital-level factors, PCI is significantly associated with the number of beds (200–399: OR 1.57, 95% CI 1.37–1.80, *P* < .001; ≥400: OR 1.38, 95% CI 1.21–1.57, *P* < .001) and hospital physician numbers (Middle: OR 1.09, 95% CI 1.00–1.19, *P* = .003; High: OR 1.41, 95% CI 1.24–1.48, *P* < .001). Public hospitals seem to perform more PCIs than private hospitals.

**Table 2:** Odds ratio for PCI on the basis of patient and hospital-level characteristics

	Total		OR (95% CI)	*P*
	PCI	No PCI		
	*N* = 6420	*N* = 5401		
**Patient characteristics**				
Sex				
Male	3984	2866	Reference	
Female	2436	2535	0.69 (0.64–0.74)	<.001
Age				
65–75	814	579	Reference	
75–79	2498	2001	0.89 (0.79–1.00)	.056
80–84	2111	1794	0.84 (0.74–0.95)	.005
≥ 85	997	1027	0.69 (0.60–0.79)	<.001
Economic status				
Low	2492	2235	Reference	
Middle	3440	2805	1.10 (1.02–1.19)	.014
High	488	361	1.21 (1.05–1.41)	.011
Type of ACS				
STEMI/ unstable angina	2451	910	Reference	
Others	3969	4491	0.33 (0.30–0.36)	<.001
Comorbidities				
Hypertension	6077	4976	1.51 (1.31–1.75)	<.001
Dyslipidemia	3838	2937	1.25 (1.16–1.34)	<.001
CCI				
No	155	177	Reference	
Mild	1593	1337	1.36 (1.08–1.71)	.008
Moderate	1976	1717	1.31 (1.05–1.65)	.017
Severe	2696	2170	1.42 (1.14–1.77)	.002
**Hospital characteristics**				
Number of beds				
≤ 199	475	561	Reference	
200–399	2408	1811	1.57 (1.37–1.80)	<.001
≥ 400	3537	3029	1.38 (1.21–1.57)	<.001
Ownership				
Public	1738	1342		
Private	4682	4059	0.89 (0.82–0.97)	.006
Hospital physician density				
Low	2228	1930	Reference	
Middle	2414	2377	0.88 (0.81–0.96)	.003
High	1778	1094	1.41 (1.28–1.55)	<.001

*PCI* percutaneous coronary intervention, *ACS* acute coronary syndromes, *STEMI* ST-elevation myocardial infarction, *CCI* Charlson Comorbidity Index, *OR* odds ratio, *CI* confidence interval

### Multilevel analyses

Three models were performed and adapted into the multilevel logistic regression model (Table 3). The null model indicated significant variations in the hospital level (ICC 0.184). The MOR that was calculated at the hospital level indicated a 2.4-times higher risk for PCI use and that at the SMA level indicated that there was no increased risk. This suggested that PCI use was attributed to the variation in the hospital level rather than the SMA level.

**Table 3:** Multilevel logistic analyses to examine variation and determinants on PCI use

	Null Model		Model 1		Model 2	
	AOR (95% CI)	*P*	AOR (95% CI)	*P*	AOR (95% CI)	*P*
**Patient characteristics**						
Sex						
Male			Reference		Reference	
Female			0.73 (0.67–0.79)	<.001	0.72 (0.67–0.79)	<.001
Age						
65–74			Reference		Reference	
75–79			0.90 (0.79–1.03)	.124	0.90 (0.79–1.03)	.124
80–84			0.86 (0.75–0.98)	.025	0.86 (0.75–0.98)	.023
≥ 85			0.70 (0.60–0.82)	<.001	0.70 (0.60–0.82)	<.001
Economic status						
Low			Reference		Reference	
Middle			0.99 (0.91–1.08)	.884	0.99 (0.91–1.08)	.900
High			1.05 (0.89–1.23)	.545	1.05 (0.90–1.24)	.535
Type of ACS						
STEMI/unstable angina			Reference		Reference	
Others			0.30 (0.28–0.34)	<.001	0.31 (0.28–0.34)	<.001
Comorbidities						
Hypertension			1.32 (1.12–1.54)	.001	1.32 (1.12–1.54)	.001
Dyslipidemia			1.20 (1.11–1.30)	<.001	1.20 (1.11–1.30)	<.001
CCI						
No			Reference		Reference	
Mild			1.21 (0.95–1.54)	.131	1.21 (0.95–1.55)	.122
Moderate			1.18 (0.93–1.51)	.172	1.18 (0.93–1.51)	.171
Severe			1.21 (0.95–1.55)	.118	1.21 (0.95–1.55)	.117
**Hospital characteristics**						
Number of beds						
≤ 199					Reference	
200–399					1.53 (1.00–2.34)	.050
≥ 400					1.11 (0.60–2.05)	.740
Ownership						
Public					Reference	
Private					1.37 (0.97–1.94)	.075
Physician density						
Low					Reference	
Middle					1.21 (0.75–1.95)	.432
High					2.05 (1.16–3.64)	.014
**δ**^**2**^						
SMA level	<.001		<.001		<.001	
Hospital level	0.744		0.640		0.541	
**ICC**						
SMA level	<.001		<.001		<.001	
Hospital level	0.184		0.163		0.141	
**PCV (%)**						
SMA level	Reference		–		–	
Hospital level	Reference		7.3		14.7	
**MOR**						
SMA level	1.001		1.001		1.000	
Hospital level	2.425		2.347		2.266	
**AIC**	15,698.0		14,957.1		14,953.4	
**Log Likelihood**	− 7845.99		− 7463.53		− 7456.7	

*PCI* percutaneous coronary intervention, *ACS* acute coronary syndromes, *STEMI* ST-elevation myocardial infarction, *SMA* secondary medical area, *CCI* Charlson Comorbidity Index, *AOR* adjusted odds ratio, *CI* confidence interval, *ICC* intraclass correlation coefficient, *MOR* median odds ratio, *AIC* Akaike’s information criterion

In model 1, a significant association with a lower proportion of PCI was observed among women (AOR 0.73, 95% CI 0.67–0.79, *P* < .001) and patients aged ≥80 years (80–84: AOR 0.86, 95% CI 0.75–0.98, *P* = .025; ≥80: AOR 0.70, 95% CI 0.60–0.82, *P* < .001). Patients with other coronary syndromes were much less likely to have PCI than those with STEMI or unstable angina (AOR 0.30, 95% CI 0.28–0.34, *P* < .001). A significant increase in PCI use was shown among patients with hypertension and dyslipidemia. Economic status was no longer statistically significant when the clustering effects of hospital and SMA were controlled. A slight decrease was found within the hospital-level variance (ICC 0.163). Similarly, a slight decrease was observed in hospital-level variance (MOR 2.347).

**Fig. 2:**
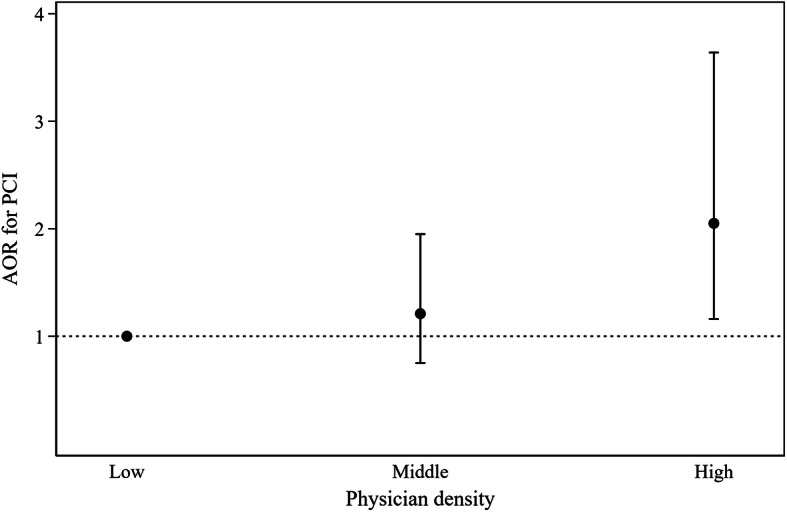
Adjusted odds ratios for the PCI for physician density after covariate adjustment in model 2. PCI, percutaneous coronary intervention

In model 2, with the introduction of hospital-level factors, there was a significant decrease in hospital-level factors for ICC (hospital level: 0.141) and MOR (hospital level: 2.266), resulting in an obvious proportional change in cluster variance that was observed at the hospital level (PCV 14.7%). By examining the results of fixed-effect estimation, women (AOR 0.72, 95% CI 0.67–0.79, *P* < .001) and patients aged ≥80 years (80–84: AOR 0.86, 95% CI 0.75–0.98, *P* = .023; ≥80: AOR 0.70, 95% CI 0.60–0.82, *P* < .001) had a significant association with lower PCI use. Patients with coronary syndrome other than STEMI or unstable angina had a lower possibility for receiving PCI (AOR 0.31, 95% CI 0.28–0.34, *P* < .001). Patient status of hypertension, dyslipidemia had significant positive relationship with PCI use. Hospitals with high physician density (AOR 2.05, 95% CI 1.16–3.64, *P* = .014) (Fig. 2) had an increased PCI use. By assessing the goodness-of-fit, the model showed better improvement than that of the previous models because of the smaller log-likelihood and AIC values.

### Sensitivity analyses

The results of sensitivity analyses are shown in Table 4. The results for model 3 with continuous CCI scores, those for model 4 with continuous age, and those for model 5 with economic status quartile categories showed a similar MOR as was estimated using our random effect models for model 2.

**Table 4:** Sensitivity analyses

	Model 3		Model 4		Model 5	
	AOR (95% CI)	*P*	AOR (95% CI)	*P*	AOR (95% CI)	*P*
**Patient characteristics**						
Sex						
Male	Reference		Reference		Reference	
Female	0.73 (0.67–0.79)	<.001	0.73 (0.67–0.79)	<.001	0.72 (0.66–0.78)	<.001
Age Category						
65–74	Reference				Reference	
75–79	0.91 (0.79–1.03)	.137			0.90 (0.79–1.03)	.125
80–84	0.86 (0.75–0.98)	.027			0.86 (0.75–0.98)	.022
≥ 85	0.71 (0.61–0.82)	<.001			0.70 (0.60–0.81)	<.001
Age (continuous)			0.98 (0.97–0.99)	<.001		
Economic status						
Very low					Reference	
Low	Reference		Reference		0.93 (0.81–1.06)	.274
Middle	0.99 (0.91–1.08)	0.899	1.00 (0.92–1.09)	.962	0.95 (0.84–1.07)	.376
High	1.05 (0.90–1.24)	0.520	1.06 (0.90–1.24)	.508	1.00 (0.83–1.20)	.997
Type of ACS						
STEMI/unstable angina	Reference		Reference		Reference	
Others	0.31 (0.28–0.34)	<.001	0.31 (0.28–0.34)	<.001	0.31 (0.28–0.34)	<.001
Comorbidities						
Hypertension	1.32 (1.12–1.55)	.001	1.31 (1.12–1.54)	.001	1.32 (1.12–1.55)	.001
Dyslipidemia	1.20 (1.11–1.30)	<.001	1.20 (1.11–1.30)	<.001	1.20 (1.11–1.30)	<.001
CCI						
No			Reference		Reference	
Mild			1.21 (0.95–1.54)	.130	1.21 (0.95–1.55)	.120
Moderate			1.18 (0.93–1.50)	.183	1.18 (0.93–1.51)	.169
Severe			1.20 (0.95–1.53)	.131	1.22 (0.95–1.55)	.114
(continuous)	1.01 (0.99–1.02)	0.379				
**Hospital characteristics**						
Number of beds						
≤ 199	Reference		Reference		Reference	
200–399	1.52 (1.00–2.34)	0.052	1.53 (1.00–2.33)	0.052	1.53 (1.10–2.55)	0.050
≥ 400	1.11 (0.60–2.04)	0.743	1.11 (0.60–2.04)	0.745	1.11 (0.60–2.05)	0.742
Ownership						
Public	Reference		Reference		Reference	
Private	1.37 (0.97–1.94)	0.078	1.37 (0.97–1.94)	0.075	1.37 (0.97–1.94)	0.075
Number of physicians						
Low	Reference		Reference		Reference	
Middle	1.21 (0.75–1.95)	0.438	1.21 (0.75–1.95)	0.432	1.21 (0.75–1.96)	0.430
High	2.04 (1.15–3.64)	0.014	2.05 (1.16–3.63)	0.014	2.06 (1.16–3.65)	0.014
**δ**^**2**^						
SMA level	<.001		<.001		<.001	
Hospital level	0.545		0.541		0.543	
**ICC**						
SMA level	<.001		<.001		<.001	
Hospital level	0.142		0.141		0.142	
**MOR**						
SMA level	1.000		1.000		1.001	
Hospital level	2.270		2.266		2.269	
**AIC**	14,951.3		14,947.8		14,954.2	
**Log Likelihood**	− 7457.652		− 7455.895		−7456.101	

*PCI* percutaneous coronary intervention, *ACS* acute coronary syndromes, *STEMI* ST-elevation myocardial infarction, *SMA* secondary medical area, *CCI* Charlson Comorbidity Index, *AOR* adjusted odds ratio, *CI* confidence interval, *ICC* intraclass correlation coefficient, *MOR* median odds ratio, *AIC* Akaike’s information criterion

## Discussion

Our study demonstrates a significant variation (MOR 2.266) in the use of PCI among hospitals, although such variation was not observed across secondary medical areas (SMAs). We found this variation took place at hospitals with high physician density. Patients’ baseline characteristics, including sex, age and comorbidities, were the determinants of PCI use. Our results could provide some useful information as the basis of decision for the allocation of acute healthcare resources and the management strategies for older patients.

Although SMA-level variation was not statistically and significantly observed in our models, we noticed that acute healthcare resources are abundantly located in metropolitan areas. Fukuoka-Itoshima and Kitakyushu are considered as metropolitan areas—densely populated urban cities in Fukuoka Prefecture. In our results, hospitals with a sufficient capacity to perform PCI were concentrated in these two SMAs. However, PCI rates in these two cities are among the lowest in Fukuoka Prefecture. Most of areas with high PCI rates are those with low population density but high number of full-time physicians (for example, Yame-Chikugo and Iizuka). At the macro level, healthcare allocation is evaluated on the basis of the population’s demographic characteristics or location (urban or rural area) [36]. However, our results showed a reversed allocation, meaning the allocation for full-time physician in low population density areas needs to be recalculated to achieve an equitable and sustainable healthcare system. In addition, Japan has a higher proportion of PCI among older adults than other countries [12]. A previous research reported that PCI rate in Fukuoka Prefecture was one of the highest in Japan (ranked 4th among 47 prefectures), and such a high rate of PCI might indicate the performance was more than needed [17]. In this circumstance, the performance of PCI among the highest PCI rate areas in Fukuoka Prefecture, especially in areas with high PCI rate, thus might indicate an oversupply.

In our random effect model, we found hospital-level variation in PCI use. By assessing some hospital characteristics, we found that patients with PCI status were frequently reported in hospitals with a high physician density. This is consistent with the previous studies demonstrating that the difference in the number of physicians per hospital could have explained the hospital-level variation in PCI use [19, 37]. Additionally, hospitals with high physician density have a tendency to oversupply medical services as reported in a systematic review [38]. As an elective procedure, the decision to receive PCI is highly influenced by physicians’ preferences and recommendations during treatment process. A previous study reported the financial benefits for hospitals despite unremarkable post-operative survival outcome among patients [16]. Considering the unclear clinical outcomes and economic benefits, it is plausible to infer that hospitals, especially those with more physician allocations, could be motivated to perform more PCI due to profitable benefits from financial incentives in the short term. In order to allocate healthcare resources equitably, health policy constraints could be focused on the supply side and a more balanced number of physicians and beds can be taken into consideration [39].

The oversupply might originate from supplier-induced demand from hospitals. Supplier-induced demand is not equal to inappropriate medical procedures because supplier-induced demand is usually happened in situations involving appropriate procedures, in which patients have great freedom of choice. We did not attempt to identify the medical appropriateness of PCI use in this study. In our study, all patients are potentially subject to PCI recommendation, and they have the final say whether to undergo PCI or not. However, patients’ decision-making would be heavily influenced by physicians’ recommendations due to the information asymmetry, thus resulting in supplier-induced demand, which was reflected in the high PCI rates in hospitals with comparatively high physician numbers.

Furthermore, our findings showed that, at the patient level, women and patients aged over 80 years old were less likely to the use of PCI, however, PCI use was not significantly related to economic status. Our results were not consistent with those of other studies that reported that patients with a low economic status had a lower probability of undergoing PCI procedures [40–42]. This may be because of the co-payment policy for the Japanese Late Elders’ Health Insurance scheme. Under this scheme, older patients’ out-of-pocket payments for the use of PCI are capped because of the high medical cost of PCI [14, 43]. From this standpoint, Japanese universal healthcare system could provide an equitable access to acute healthcare services with high medical cost. Additionally, in our results, patients aged over 80 years old was observed to have a lower possibility to receive PCI. This can be explained by the presence of complex comorbidities, making the prognosis unclear for those older patients [12], and the expectations of patients aged over 80 years old could not be met based on the existing clinical and economical evidence. Therefore, in order to promote a more equitable access to acute healthcare services, among older patients, a treatment process guideline on PCI targeting older patients should be explored. Experts from multidisciplinary teams are anticipated to work together to evaluate relevant PCI indications for older adults by considering their comorbidities and needs in this guideline.

The study also has several limitations. First, we obtained the data from the insurance association, and it was not specifically designed for clinical research purposes. Detailed clinical indications on the severity of diseases was not accessible; therefore, we could not rule out whether patients who received PCI were already in a worse condition. Instead, we distinguished patients’ STEMI and unstable angina statuses during admission as a covariate in the models. Nevertheless, the inclusion of more detailed clinical indications in statistical models, is recommended for researchers in the future study. We also adjusted for the patients’ health statuses using their comorbidity status as a covariate. Second, the number of cardiovascular beds and cardiologists/operators were not available in our database. Instead, we used the total number of beds and physician density at the hospitals to present the characteristics at the hospital level. Because PCI is mainly performed in a hospital setting with acute care, the total number of beds and physician density can reflect health care resources that are related to acute care settings, including PCI. Furthermore, coding errors and misclassification might also be possible, which are likely to be random errors with little influence on the statistical inferences.

Despite of these limitations, our study has some strengths. First, to the best of our knowledge, this study is one of the first studies that examined small-area and hospital variations in the use of PCI in Japan. Second, our multilevel models included both patient and hospital characteristics, which could quantitatively better assess the determinants on PCI use. Third, the sample-size is large enough to obtain statistically robust findings. It is because the insurance claim database we used covered more than 600,000 insured people in Fukuoka Prefecture, and the penetration rate was as high as 98.6%.

## Conclusions

We found significant hospital-level variation in the use of PCI, while variation was failed to observe in the small area. The observed variation is not fully explained by patient baseline characteristics, but explained mostly by hospital physician density. These findings suggests that patients in the small area have an equitable ability to attain healthcare service. However, the economic incentives for PCI use arose from both hospital- and patient-level characteristics should not be ignored. To make a more equitable access to obtain healthcare service, a balanced number of physicians and beds and a treatment process guideline on PCI targeting older patients should be explored.

## Data Availability

The data are available upon request.
